# The neurorepellent, Slit2, prevents macrophage lipid loading by inhibiting CD36-dependent binding and internalization of oxidized low-density lipoprotein

**DOI:** 10.1038/s41598-021-83046-x

**Published:** 2021-02-11

**Authors:** Bushra Yusuf, Ilya Mukovozov, Sajedabanu Patel, Yi-Wei Huang, Guang Ying Liu, Emily C. Reddy, Marko Skrtic, Michael Glogauer, Lisa A. Robinson

**Affiliations:** 1 Program in Cell Biology, The Hospital for Sick Children Research Institute, Toronto, ON M5G 1X8 Canada; 2 Institute of Medical Science, University of Toronto, Toronto, ON M5S 2Z9 Canada; 3 Department of Dermatology and Skin Science, University of British Columbia, Vancouver, BC Canada; 4 Program in Developmental and Stem Cell Biology, The Hospital for Sick Children Research Institute, Toronto, ON M5G 1X8 Canada; 5 Faculty of Dentistry, Matrix Dynamics Group, University of Toronto, Toronto, ON M5G 1G6 Canada; 6 Department of Paediatrics, University of Toronto, Toronto, ON M5G 1X8 Canada

**Keywords:** Super-resolution microscopy, Actin, Mechanisms of disease

## Abstract

Atherosclerosis is characterized by retention of modified lipoproteins, especially oxidized low density lipoprotein (oxLDL) within the sub-endothelial space of affected blood vessels. Recruited monocyte-derived and tissue-resident macrophages subsequently ingest oxLDL by binding and internalizing oxLDL via scavenger receptors, particularly CD36. The secreted neurorepellent, Slit2, acting through its transmembrane receptor, Roundabout-1 (Robo-1), was previously shown to inhibit recruitment of monocytes into nascent atherosclerotic lesions. The effects of Slit2 on oxLDL uptake by macrophages have not been explored. We report here that Slit2 inhibits uptake of oxLDL by human and murine macrophages, and the resulting formation of foam cells, in a Rac1-dependent and CD36-dependent manner. Exposure of macrophages to Slit2 prevented binding of oxLDL to the surface of cells. Using super-resolution microscopy, we observed that exposure of macrophages to Slit2 induced profound cytoskeletal remodeling with formation of a thick ring of cortical actin within which clusters of CD36 could not aggregate, thereby attenuating binding of oxLDL to the surface of cells. By inhibiting recruitment of monocytes into early atherosclerotic lesions, and the subsequent binding and internalization of oxLDL by macrophages, Slit2 could represent a potent new tool to combat individual steps that collectively result in progression of atherosclerosis.

## Introduction

A key feature of atherosclerosis is the retention of modified lipoproteins within the sub-endothelial space of the vascular wall. Oxidation of low-density lipoproteins is thought to be the most significant modification, as it causes endothelial cell dysfunction and promotes recruitment of monocyte-derived macrophages to the intima of the vessel^1^. The uptake of oxidized LDL (oxLDL) by macrophages is initially protective, but over time, excessive engulfment of cholesterol induces formation of lipid-laden foam cells, which undergo necrosis and release pro-inflammatory stimuli, exacerbating atherogenesis^1^. In human and mouse macrophages, the binding and internalization of oxLDL is predominantly mediated by the scavenger receptor, CD36^2,3^. These events require dynamic re-arrangement of the actin cytoskeleton through modulation of Rho-family GTPase activity^3–6^.

Central to the initiation of atherosclerosis is the recruitment of circulating monocytes to the injured vessel wall^1^. Monocytes are recruited by secreted chemoattractants that are locally produced^7,8^. Indeed, human subjects with polymorphisms in the chemokine receptors, CX_3_CR1 and CCR2, exhibit less chemotactic migration of their monocytes to the cognate chemokine ligands, and are highly protected from cardiovascular morbidity and mortality^9–11^. Mice lacking CCR2, CCR5, and CX_3_CR1 show reduced monocyte infiltration into vascular lesions, and triple deletion of all three receptors confers additive protection from atherosclerosis^12^. Thus, simultaneous blockade of monocyte trafficking in response to diverse chemotactic cues may prove therapeutically beneficial.

The Slit family of secreted proteins act through their cell-surface Roundabout (Robo) receptors to repel migrating neurons in the developing central nervous system^13^. More recently, we and others have shown that secreted Slit2 interacts with Robo-1 on the surface of monocytes, neutrophils, lymphocytes, and vascular smooth muscle cells to prevent activation of Rho family-GTPases, thereby inhibiting migration of these cell types, all of which are intimately involved in atherogenesis, to diverse chemotactic cues^14–18^. We observed that Slit2 can inhibit not only recruitment of circulating monocytes, but their post-adhesion stabilization on activated vascular endothelial cells^14^. We further found that peripheral blood mononuclear cells from patients with coronary artery disease expressed lower surface levels of Robo-1, and that in atherosclerosis-prone mice, Slit2 inhibited recruitment of circulating monocytes into nascent atherosclerotic lesions^14^. How Slit2 affects the uptake of modified lipoproteins by macrophages has previously not been explored.

We report here that primary human and murine macrophages express the Slit2 receptor, Robo-1, and that Slit2 inhibited macrophage uptake of oxLDL in a Rac1-dependent and CD36-dependent manner. We further found that exposure of macrophages to Slit2 induced formation of actin-rich regions within the cortex that excluded CD36, resulting in less binding of oxLDL to the cell surface. Together these data support a novel role for Slit2 in decreasing macrophage lipid burden.

## Results

### NSlit2 inhibits uptake of oxLDL by macrophages

**Figure 1:**
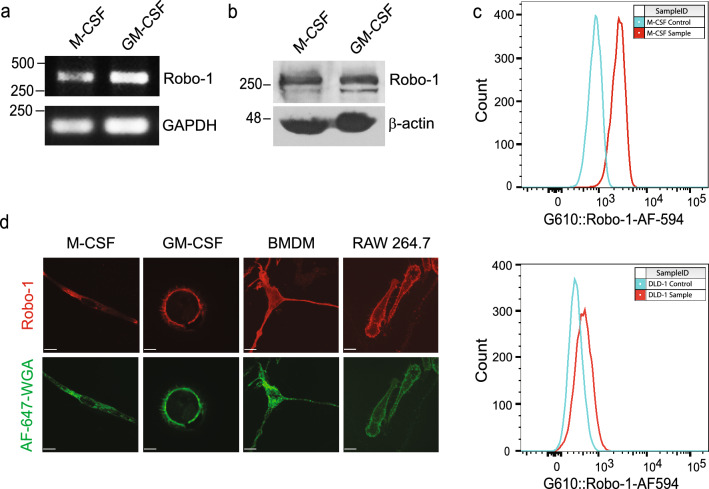
Human and murine macrophages express the Slit2 receptor, Robo-1. (**a**), Human peripheral blood mononuclear cells (PBMC) were isolated and incubated with M-CSF or GM-CSF to generate M-CSF- or GM-CSF-induced macrophages, respectively. RT-PCR was performed using specific primers for Robo-1 and GAPDH. (**b**), Lysates were collected from M-CSF- and GM-CSF-induced human macrophages and immunoblotting was performed using antibodies specifically detecting Robo-1 or ß-actin. (**c**), M-CSF-induced human macrophages (top) and DLD-1 cells (bottom) were first incubated with viability dye followed by AF-594-conjugated secondary Ab (Control, blue peak), or anti-Robo-1 Ab and AF-594-conjugated secondary Ab (Sample, red peak). Data from live, single cells was acquired using an LSRII flow cytometer (BD Biosciences-US) with FACSDiva software (BD Biosciences-US) and analyzed with FlowJo v10 (BD Biosciences-US). (**d**), M-CSF- and GM-CSF-induced human macrophages, murine BMDM and RAW264.7 macrophages were fixed and incubated with anti-Robo-1 Ab, followed by Dylight549-conjugated secondary Ab (red) and AF-647-conjugated wheat germ agglutinin (pseudocoloured green). Cells were imaged using a spinning disk confocal microscope at 63× magnification. Scale bar, 10 μm.

**Figure 2:**
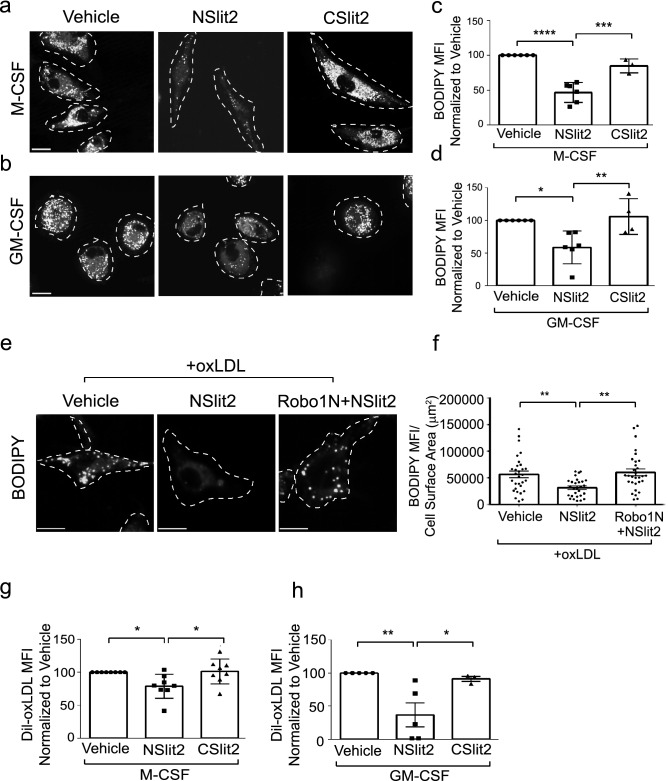
NSlit2 inhibits uptake of oxLDL by macrophages. (**a**), M-CSF-induced human macrophages were incubated with vehicle, NSlit2 or CSlit2, then incubated with oxLDL for 24 h. Internalized oxLDL was labeled with BODIPY. Representative images were obtained using a spinning disk confocal microscope at 63× magnification. Cell borders are indicated by dashed lines. Scale bar, 10 μm. (**b**), Experiments were performed as in (**a**) using GM-CSF-induced human macrophages. (**c**), Experiments were performed as in (**a**). Cells were then detached and BODIPY mean fluorescence intensity (MFI) was measured by flow cytometry. (**d**), Experiments were performed as in (**b**). Cells were then detached and BODIPY MFI was measured by flow cytometry. (**e**), M-CSF-induced human macrophages were preincubated with vehicle, NSlit2 or Robo1N + NSlit2 followed by incubation with oxLDL for 2 h. Internalized oxLDL was labeled with BODIPY. Representative images using a spinning disk confocal microscope at 40× magnification. Cell borders are indicated via dashed lines. Scale bar, 10 μm. (**f**), Quantification of experiments performed in (**e**). Individual dots correspond to single cells. Mean ± SEM from 4 independent experiments. (**g**), M-CSF-induced human macrophages were incubated with vehicle, NSlit2 or CSlit2, followed by incubation with DiI-oxLDL for 20 min. Cells were washed, detached and internalized DiI-oxLDL MFI was measured by flow cytometry. Data are the mean ± SEM of 8 independent experiments. (**h**), Experiments were performed as in (**g**) using GM-CSF-induced macrophages. Data are the mean ± SEM of 3 to 5 independent experiments. For (**c**), (**d**), samples were acquired using a Gallios flow cytometer (Beckman Coulter-US) with Kaluza Acquisition software (Beckman Coulter-US) and analyzed with FlowJo v10 (BD Biosciences-US). For (**c**), (**d**), (**f**)–(**h**), *p < 0.05, **p < 0.01, ***p < 0.001, ****p < 0.0001 were determined by one-way ANOVA using Tukey’s post hoc test.

To investigate whether Slit2 modulates macrophage oxLDL uptake, we first determined Robo-1 expression in human and murine macrophages. Robo-1 mRNA and protein were detected in M-CSF- and GM-CSF-induced human macrophages (Fig. 1a,b; Supplementary Fig. S1a,b). Using flow cytometry, anti-Robo-1 Ab detected Robo-1 in primary human M-CSF-derived macrophages but not in DLD-1 cells, which lack Robo-1^19,20^ (Fig. 1c). Robo-1 was also detected via immunofluorescence microscopy in M-CSF- and GM-CSF-induced human macrophages, murine bone marrow derived macrophages (BMDM) and murine RAW264.7 macrophages (Fig. 1d; Supplementary Fig. S1c,d). We next examined the effects of Slit2 on macrophage internalization of oxLDL over 24 h, using two different approaches, namely immunofluorescence microscopy and flow cytometry. Vehicle-treated M-CSF- and GM-CSF-induced human macrophages robustly internalized oxLDL (Fig. 2a–d). Incubation of macrophages with bio-active NSlit2, but not bio-inactive CSlit2, significantly inhibited oxLDL uptake (Fig. 2a–d).

To further verify these observations, we biochemically measured lipid uptake in macrophages. M-CSF- and GM-CSF-induced human macrophages incubated with oxLDL displayed significantly higher lipid droplet-associated cholesteryl esters within the cells compared to cells incubated with vehicle alone (Supplementary Fig. S2a,b). In contrast, incubation with bio-active NSlit2 significantly decreased intracellular cholesteryl ester content (Supplementary Fig. S2a,b). Taken together, these data indicate that macrophage exposure to NSlit2 decreases intracellular lipid uptake.

To determine whether the inhibitory effect of NSlit2 on lipid uptake is mediated by its receptor, Robo-1, we used Robo1N, a soluble N-terminal fragment of Robo-1, known to inhibit binding of Slit2 to Robo-1, thereby blocking any downstream effects^21–23^. In the presence of NSlit2, macrophages internalized significantly less oxLDL (Fig. 2e, f). The effects of NSlit2 were reversed by incubation with antagonistic Robo1N (Fig. 2e, f). Together, these results suggest that NSlit2 inhibits the internalization of oxLDL in human and murine macrophages in a Robo-1-dependent manner.

The overall uptake of oxLDL by macrophages reflects the balance between lipid internalization and efflux. To determine how NSlit2 affects lipid internalization, we incubated M-CSF- and GM-CSF-induced macrophages with fluorescently-labeled oxLDL for 20 min. In the presence of NSlit2, macrophage internalization of oxLDL was significantly diminished (Supplementary Fig. S2c,d). We verified these findings using flow cytometry and again observed that bio-active NSlit2, but not bio-inactive CSlit2, attenuated internalization of oxLDL (Fig. 2g, h). Similarly, exposure to NSlit2 inhibited internalization of oxLDL by murine BMDM (Supplementary Fig. S2e,f).

We next examined the effects of NSlit2 on cholesterol efflux using the cholesterol acceptor, high-density lipoprotein (HDL). We did not observe any differences in cholesterol efflux between macrophages incubated with bio-active NSlit2 or those incubated with inactive controls CSlit2 and ΔD2-Slit2 (ΔD2), which lacks the Robo-1-binding domain^19,21,24^ (Supplementary Fig. S2g). Together, these data suggest that NSlit2 attenuates sustained accumulation of oxLDL in macrophages, at least in part, by inhibiting lipid internalization.

### Inhibition of oxLDL uptake by macrophages is CD36-dependent

**Figure 3:**
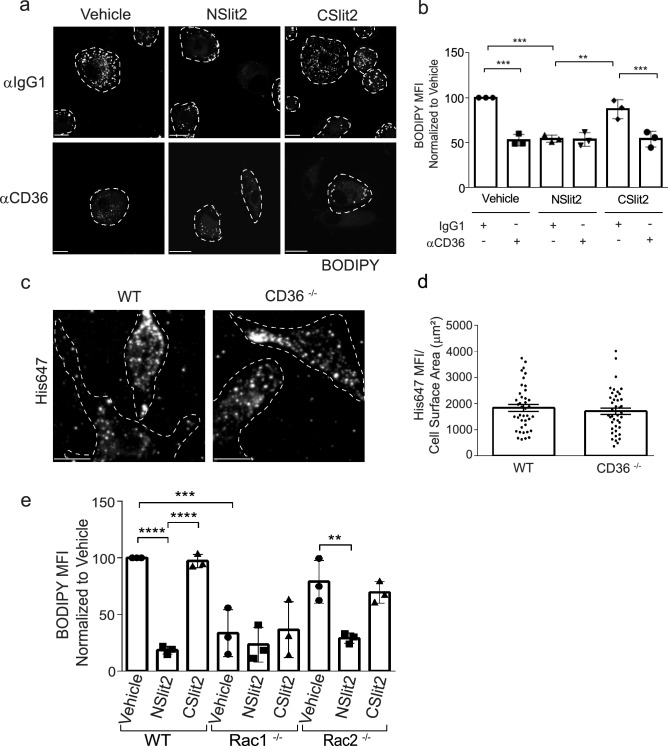
NSlit2-mediated inhibition of oxLDL uptake by macrophages is CD36- and Rac1-dependent. (**a**), M-CSF-induced human macrophages were incubated with anti-CD36 Ab or with an isotype-matched IgG1 Ab for 30 min, followed by incubation with vehicle, NSlit2, or CSlit2. Macrophages were then incubated with oxLDL for 24 h and labeled with BODIPY to visualize intracellular lipid droplets. Representative images were acquired using a spinning disk confocal microscope. BODIPY-labeled oxLDL puncta are shown pseudocoloured white and cell borders are indicated via dashed lines. Scale bar, 10 μm. (**b**), Quantification of experiments performed in (**a**). BODIPY MFI was quantified using ImageJ Software and 20–30 cells per experimental group were analyzed. Data are the mean ± SEM of 3 independent experiments. (**c**), BMDM from wild-type (WT) and CD36^−/−^ mice were incubated with NSlit2 at 4 °C followed by incubation with AF-647-conjugated anti-His Ab at 4 °C. Cells were fixed and representative images of cell surface labeling were acquired using a spinning disk confocal microscope. Dashed lines indicate cell borders. Scale bar, 10 μm. (**d**), Quantification of (**c**). His647 MFI was quantified using Volocity Software and 20–30 cells were analyzed for each experimental condition. Individual dots correspond to single cells pooled from 4 independent experiments. Vertical bars represent the mean ± SEM of the pooled data. Comparison was determined by an unpaired, t-test, and was not significant. (**e**), BMDM from WT, Rac1^−/−^, and Rac2^−/−^ mice were incubated with vehicle, NSlit2, or CSlit2, then with oxLDL for 24 h. Internalized oxLDL was labeled with BODIPY and measured as described in (**b**). BODIPY MFI was measured in 40–50 cells per experimental group. Data are the mean ± SEM of 3 independent experiments. For (**b**) and (**e**), **p < 0.01, ***p < 0.001, ****p < 0.0001 were determined by one-way ANOVA using Tukey’s post hoc test.

Since the scavenger receptor, CD36, has been shown to promote more than 50–60% of internalization of oxLDL by macrophages^3^, we tested the effects of NSlit2 on CD36-mediated oxLDL uptake. Macrophages were incubated with a blocking Ab directed to CD36 or an isotype-matched control IgG1 Ab, followed by incubation with oxLDL. In vehicle-treated macrophages, incubation with IgG1 Ab led to robust foam cell formation (Fig. 3a, b) while incubation with anti-CD36 Ab significantly decreased lipid accumulation (Fig. 3a, b). Macrophages incubated with CSlit2 and IgG1 Ab displayed comparable lipid accumulation to vehicle-treated cells (Fig. 3a, b). Macrophages exposed to NSlit2 and incubated with IgG1 Ab demonstrated significantly less oxLDL uptake relative to vehicle-treated macrophages incubated with IgG1 Ab (Fig. 3a, b). No further decrease in lipid accumulation was observed in macrophages incubated with both NSlit2 and anti-CD36 Ab (Fig. 3a, b).

In principle, NSlit2 could cause the observed inhibition of oxLDL uptake by directly binding to CD36. To investigate this possibility, we incubated human macrophages with blocking anti-CD36 Ab prior to incubation with fluorescently-labeled NSlit2. Binding of NSlit2 to cells incubated with anti-CD36 Ab was comparable to that of cells incubated with isotype control Ab (Supplementary Fig. S3a,b). To further verify these findings, we examined binding of fluorescently-labeled NSlit2 to the surface of BMDM from CD36-deficient (CD36^−/−^) and wild-type (WT) mice. We detected similar binding of NSlit2 to both CD36^−/−^ and WT macrophages (Fig. 3c, d; Supplementary Fig. S3c–e). We also confirmed that levels of Robo-1 are similar in CD36^−/−^ and WT macrophages (Supplementary Fig. S3f). Taken together, these results suggest that NSlit2 may inhibit uptake of oxLDL by inhibiting CD36-mediated oxLDL uptake and that this effect is not likely due to physical interaction between NSlit2 and CD36.

### NSlit2-mediated inhibition of oxLDL uptake is Rac1-dependent

Engagement of cell surface CD36 by oxLDL induces activation of Rac through activation of the Rac guanine nucleotide exchange factor (GEF), Vav^25,26^. Furthermore, internalization of oxLDL by CD36 is a dynamic actin-driven process that involves activation of Rac1^3,26^. To investigate the role of Rac in Slit2-mediated inhibition of oxLDL uptake, we performed lipid loading assays in BMDM from WT, Rac1-deficient (Rac1^−/−^) and Rac2-deficient (Rac2^−/−^) mice^27,28^. WT BMDM exposed to vehicle or bio-inactive CSlit2 showed robust and comparable accumulation of cholesterol esters (Fig. 3e). Incubation of WT BMDM with NSlit2 significantly decreased cholesterol ester accumulation (Fig. 3e). Overall, BMDM isolated from Rac1^−/−^ mice displayed less cholesterol ester accumulation than WT BMDM (Fig. 3e). Rac1^−/−^ BMDM incubated with NSlit2 did not show further impairment in cholesterol ester accumulation compared to Rac1^−/−^ BMDM incubated with vehicle or CSlit2 (Fig. 3e). Vehicle-treated Rac2^−/−^ BMDM showed a trend toward lower cholesterol ester accumulation relative to vehicle-treated WT BMDM, although this difference was not significant (Fig. 3e). Uptake of oxLDL was comparable in Rac2^−/−^ BMDM exposed to vehicle or CSlit2 (Fig. 3e). Incubation of Rac2^−/−^ BMDM with NSlit2 resulted in significantly lower cholesterol ester accumulation (Fig. 3e). Taken together, these results suggest that Slit2-mediated inhibition of cholesterol ester accumulation in macrophages is dependent on Rac1, but not Rac2.

### NSlit2 decreases binding of oxLDL to macrophages

**Figure 4:**
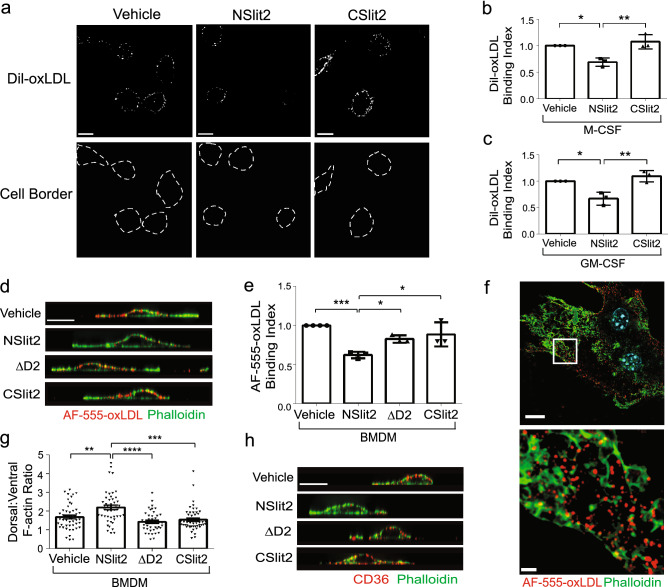
NSlit2 inhibits binding of oxLDL to the surface of macrophages by depleting actin-poor zones in which CD36 clusters. (**a**), M-CSF-induced human macrophages were incubated with vehicle, NSlit2, or CSlit2, followed by a 1 min incubation with DiI-oxLDL at 37 °C. Cells were washed to remove unbound DiI-oxLDL and then fixed. Representative images were obtained using a spinning disk confocal microscope. Cell borders are indicated via dashed lines (bottom row). Scale bar, 10 μm. (**b**), Quantification of (**a**). For each experimental condition, the MFI of bound DiI-oxLDL was quantified for 20–40 cells using Image J Software. Data are the mean ± SEM of 3 independent experiments. (**c**), Experiments were performed as in (**b**), using GM-CSF-induced macrophages. Data are the mean ± SEM of 3 independent experiments. (**d**) BMDM were incubated with vehicle, NSlit2, CSlit2, or ΔD2, then incubated with AF-555-conjugated oxLDL (red) for 1 min at 37 °C. Cells were fixed and incubated with AF-488-conjugated phalloidin (green). Representative XZ slices were obtained using a spinning disk confocal microscope. Scale bar, 10 μm. (**e**), Quantification of experiments performed in (**d**). For each experimental condition, the MFI of bound AF-555-conjugated oxLDL was quantified for 20–40 cells using Image J Software. Data are the mean ± SEM of 3–4 independent experiments. (**f**), Experiments were performed as in (**d**), and super resolution, Structured Illumination Microscopy (SIM) used to obtain representative XY optical slices. Scale bars; 15 μm (top), 2 μm (bottom). (**g**), Quantification of experiments performed in (**d**). The dorsal:ventral F-actin ratio was quantified using ImageJ Software to measure the fluorescence intensity of AF-488-conjugated phalloidin and 30–40 cells were analyzed for each experimental condition. Mean ± SEM from 4 to 5 independent experiments. (**h**)**,** BMDM were incubated with vehicle, NSlit2, CSlit2 or ΔD2, then fixed and incubated with anti-CD36 Ab followed by AF-647-conjugated anti-rat IgG (pseudocoloured red) and AF-488-conjugated phalloidin (green). Representative XZ slices were obtained using a spinning disk confocal microscope. Scale bar, 10 μm. For (**b**), (**c**), (**e**), (**g**) *p < 0.05, **p < 0.01, ***p < 0.001, ****p < 0.0001 were determined by one-way ANOVA using Tukey’s post hoc test.

We reasoned that NSlit2 could decrease the amount of cholesterol stored in lipid droplets by inhibiting binding of oxLDL to macrophages. To test this notion, we incubated macrophages with fluorescently-labeled oxLDL for 1 min—a time point at which internalization of oxLDL is negligible^29^—then examined cell surface-associated oxLDL (Fig. 4a–f). Incubation with bio-inactive CSlit2 had no effect on cell surface binding of oxLDL (Fig. 4a–c), whereas NSlit2 inhibited binding of oxLDL to the surface of both M-CSF- and GM-CSF-induced human macrophages (Fig. 4a–c). Similarly, in the presence of bio-inactive fragments CSlit2 and ΔD2, binding of oxLDL to the surface of murine BMDM was not affected (Fig. 4d, e). In the presence of NSlit2, binding of oxLDL to the surface of BMDM was inhibited (Fig. 4d, e). Taken together, these results indicate that NSlit2 attenuates binding of oxLDL to the surface of human and murine macrophages.

### NSlit2 inhibits formation of actin-poor regions in the plasma membrane within which CD36 clusters

We previously demonstrated that CD36 clusters within actin-poor regions of the plasma membrane of macrophages, and that these actin-poor regions are precisely where binding of oxLDL occurs^29^. We, therefore, questioned whether NSlit2 inhibits oxLDL binding to the surface of macrophages by disrupting actin-poor regions within which CD36 is enriched. To test this notion, we examined the distribution of oxLDL and F-actin in murine BMDM. Using super-resolution microscopy, we again observed that oxLDL preferentially bound in actin-poor regions of the dorsal surface of vehicle-treated macrophages (Fig. 4f). We next examined the effects of NSlit2 on the distribution of F-actin in macrophages. Exposure of macrophages to bio-inactive CSlit2 or ΔD2 resulted in a similar distribution of dorsal-to-ventral F-actin as observed in cells exposed to vehicle alone (Fig. 4d, g, h). Following exposure to NSlit2, BMDM demonstrated a greater density of F-actin along the dorsal surface compared to macrophages incubated with vehicle, CSlit2, or ΔD2 (Fig. 4d, g, h). Accordingly, following incubation with NSlit2, the dorsal surface of macrophages bound less oxLDL (Fig. 4d, e). In keeping with these observations, incubation of macrophages with NSlit2 induced formation of a thick ring of dorsal F-actin, thereby inhibiting clustering of CD36 on the dorsal cell surface (Fig. 4h). Overall, these results demonstrate that NSlit2 inhibits binding of oxLDL to the surface of macrophages by promoting formation of actin-rich zones which exclude CD36.

## Discussion

We report here that human and mouse macrophages express the Slit2 receptor, Robo-1. Our findings are in keeping with those of others who similarly demonstrate expression of Robo-1 in primary and cultured murine macrophages^30–32^. Although a variety of surface markers, including CD14 and CD16, are differentially expressed in M-CSF- versus GM-CSF-induced macrophages, we found that Robo-1 was expressed in both^33^.

We used immunofluorescence microscopy and flow cytometry to detect fluorescently labeled intracellular lipid. By both approaches, we found that exposure of macrophages to NSlit2 inhibited oxLDL uptake. To further confirm our observations, we also used a biochemical method for sensitive quantization of lipid uptake by macrophages. By this method, we again observed that M-CSF- and GM-CSF-induced human macrophages incubated with oxLDL displayed significantly higher intracellular, lipid droplet-associated cholesteryl esters than those incubated with vehicle alone, and that incubation with NSlit2 significantly decreased intracellular cholesteryl ester content. Interestingly, we did not observe a statistical difference between groups treated with NSlit2 alone and those treated with NSlit2 and oxLDL, possibly underscoring the inhibitory effect of NSlit2 on the uptake of oxLDL. While we observed a trend towards lower intracellular cholesteryl ester content in cells exposed to NSlit2 in comparison to cells exposed to bio-inactive CSlit2, this difference did not reach significance. This may be due to the high sensitivity of the assay, which employs an enzyme-coupled reaction paired with Amplex Red reagent to detect even low amounts of cholesterol.

We observed that exposure of macrophages to NSlit2 inhibited oxLDL uptake in a Robo-1- and Rac1-dependent manner. Previous studies suggest that Slit2 does not interact directly with Rac, but rather, that binding of Slit2 to Robo-1 can result in inactivation of Rac through the actions of slit-robo GTPase activating proteins, srGAPs^14,34^. Accordingly, we recently demonstrated that murine macrophages express srGAP2, and that exposure of mouse macrophages to Slit2 causes inactivation of Rac in these cells^23^. Our results are also consistent with work demonstrating a key role for Rac-mediated signaling in foam cell formation^35^. Signaling events that follow engagement of CD36 by oxLDL and lead to ligand-receptor internalization have also been demonstrated to require the activation of Rac^3^. Our results are also consistent with work showing that Vav, an upstream GEF for Rac, enhances murine atherogenesis and foam cell formation, likely through activation of Rac^5^.

We examined the differential involvement of Rac1 vs Rac2 in lipid uptake, and observed that uptake of oxLDL by Rac1^−/−^ macrophages was greatly impaired, and additional exposure to NSlit2 did not further reduce oxLDL uptake. In contrast, although uptake of oxLDL was diminished in Rac2^−/−^ macrophages (albeit to a lesser degree than in Rac1^−/−^ macrophages), exposure to NSlit2 resulted in further inhibition of oxLDL uptake. In macrophages themselves, Rac1 and Rac2 have been shown to play distinct roles in modulating changes in cell morphology, membrane ruffling, podosome formation, phagocytosis and cell invasion^36,37^. Furthermore, in murine macrophages, Rac1 is four-fold more abundant than Rac2, which may explain the differential responses we observed and the different phenotype of Rac1^−/−^ versus Rac2^−/−^ macrophages^38^. Given this context, the effects of inhibition of Rac1 may be so pronounced that no further inhibition of oxLDL uptake by NSlit2 is possible. Taken together, our results suggest that Rac1 plays a more important role in uptake of oxLDL by macrophages than does Rac2, and further, that inhibitory effects of NSlit2 on uptake of oxLDL involve signaling pathways other than Rac2.

The Rac-mediated inhibition of macrophage lipid loading by NSlit2 that we observed is consistent with studies demonstrating a clear link between Rac and cytoskeletal patterning during particle uptake. Indeed, Rac1 is recruited to membrane ruffles of cholesterol-loaded macrophages, and shows extensive colocalization with F-actin^35^. Additionally, Flannagan et al. demonstrated that dynamic macrophage ‘probing’ is required to efficiently bind phagocytic targets and that this probing behavior and efficient binding of particles are dependent on dynamic actin polymerization and Rac1 activation^4^. Thus, it is possible that in our studies, NSlit2 also inhibited the ability of macrophages to probe their environment and engage oxLDL by inhibiting Rac activation and actin polymerization.

We observed that when CD36 was inhibited, addition of NSlit2 did not further decrease macrophage lipid content suggesting that in our studies, NSlit2 did not affect alternate cellular pathways of lipid internalization. Our results further suggest that the NSlit2-mediated decrease in lipid content is not due to direct physical blockade of CD36 by NSlit2. However, it is possible that the inhibition of oxLDL uptake induced by antibody blockade of CD36 may be the strongest inhibition that is indeed achievable, making detection of any further inhibition by NSlit2 impossible within the experimental system we used. Overall, our data are in keeping with evidence that CD36-mediated internalization of oxLDL by macrophages requires re-arrangement of actin, and that internalization of CD36 requires activation of Rac1^3,6^. Although internalization of CD36 and oxLDL require activation of JNK, Slit proteins have not been reported to inactivate JNK, making JNK inhibition an unlikely explanation for our observations^3,39,40^. Published studies indicate that CD36 physically associates with the Src kinases Fyn, Lyn and Yes and that CD36 signaling through Src kinases triggers internalization of bound ligands^41,42^. In macrophages and microglia, CD36- Src kinase signaling promotes phosphorylation of focal adhesion kinase (FAK) and FAK-associated proteins, resulting in actin remodeling^43,44^. Accordingly, Slit2 has been reported to inhibit activation of Src and FAK^17,45,46^. As such, in our studies, NSlit2-mediated inhibition of one or both of these processes could possibly contribute to the observed decrease in CD36-dependent lipid uptake by macrophages. However, we recently investigated the effects of Slit2 on Src signaling in macrophages, and did not observe any effects on activation of Src kinases^23^.

We found that exposure of macrophages to NSlit2 inhibited binding of oxLDL to the cell surface by disrupting actin zoning that favors clustering of CD36 and consequent binding of oxLDL. These findings are in accord with the observation that receptor coalescence of CD36 is required for ligand capture and downstream signal transduction^47–49^. CD36 clustering is a consequence of receptor diffusion, and is spatially controlled by the cortical cytoskeleton^49^. Jaqaman et al. reported regions of isotropic or confined motion of CD36 in macrophages, as well as regions in which the receptor’s diffusion is relatively unobstructed^49^. These differences in diffusion reflected differences in the submembranous actomyosin meshwork such that actin-delimited channels in the plasma membrane promoted receptor diffusion, collision and cluster formation^49^. Consistent with this notion, we previously reported that in response to chemokine stimulation, the cortical actin cytoskeleton of macrophages undergoes extensive remodelling, inducing formation of actin-poor regions within the plasma membrane^29^. We found that CD36 clustering occurred exclusively within these actin-poor zones, enhancing cell surface binding of oxLDL, and accelerating foam cell formation^29^. Conversely, we report here that NSlit2 induces formation of a thick actin-rich region within the plasma membrane that excludes CD36, and precludes binding and internalization of oxLDL by macrophages.

We and others have shown that Slit2-Robo-1 signaling negatively regulates migration of different leukocyte subsets—namely, monocytes, neutrophils, lymphocytes, and dendritic cells—involved in vascular inflammation^14–18^. The potentially protective effects of Slit2 extend beyond those on leukocyte trafficking. Indeed, Slit2 has been shown to inhibit migration of aortic smooth muscle cells towards inflammatory cues, and acts as a potent anti-platelet agent both in vitro and in vivo^21,50^. We recently showed that Slit2 inhibits macropinocytosis of macrophages, and in this way, can inhibit their secretion of the inflammatory chemokine, CXCL1, following exposure to inflammatory stimuli^23^. We now report here that NSlit2 inhibits oxLDL uptake and foam cell formation by macrophages. Thus, NSlit2 could potentially be harnessed as a therapeutic capable of individually attenuating each of these pro-atherogenic events, that, collectively, result in initiation and progression of atherosclerotic lesions.

## Materials and methods

### Ethics statement

Written, informed consent was obtained prior to blood draws from all healthy volunteers. All procedures were approved by the Hospital for Sick Children (REB) under protocol 1000060065 and adhered to the guidelines and regulations stated in the Hospital for Sick Children Research Ethics Board (REB) Blood Sampling Guidelines. All animal studies presented were conducted in a specific pathogen-free animal facility at the Hospital for Sick Children. The experiments were registered and approved by The Hospital for Sick Children Institutional Animal Care and Use Committee, and adhered to the approved Animal Use Protocol (AUP No. 1000043631) in accordance with the Canadian Council on Animal Care Guidelines and federal and provincial regulations/legislation.

### Reagents and antibodies

The following reagents were purchased from Thermo Fisher Scientific (Rockford, IL, USA): rabbit polyclonal anti-Robo-1 antibody (Ab; PA5-29917); BODIPY 493/503 (4,4-Difluoro-1,3,5,7,8-Pentamethyl-4-Bora-3a,4a-Diaza-s-Indacene) (D3922); FITC-, AF-488- and AF-647-conjugated Wheat Germ Agglutinin (WGA); AF-488-conjugated phalloidin; DAPI (4′,6-diamidino-2-phenylindole dihydrochloride), AF-594-conjugated donkey anti-rabbit Ab (A21207), LIVE/DEAD™ Fixable Violet Dead Cell Stain Kit (L34963), AF-555 Succinimidyl Ester (A20009), eBioscience Permeabilization Buffer 10× (00-8333-56), Amplex Red Cholesterol Assay Kit (A12216), TRIzol Reagent (15596026), SuperScript III Reverse Transcriptase (18080093), SuperScript IV VILO Master Mix (11756500) and POWER SYBR Green Master Mix (4367659). Mouse monoclonal anti-β-actin Ab (A5441) and protease inhibitor cocktail (P2714) were purchased from Sigma-Aldrich Canada (Oakville, ON, Canada). Mouse monoclonal anti-Robo-1 Ab (MAB 71181) and recombinant Robo1N protein were purchased from R&D Systems (Minneapolis, MN, USA). Rabbit polyclonal anti-Robo-1 Ab (600-401-692) and DyLight549-conjugated donkey anti-mouse Ab (610-742-002) were purchased from Rockland Immunochemicals, Inc. (Pottstown, PA, USA). 10× RIPA buffer (Ab156034), Cholesterol Efflux Assay Kit (Ab196985), mouse IgG1 [B11/6] Ab (Ab91353), mouse monoclonal anti-CD36 Ab (Ab17044) and rat anti-CD36 Ab (Ab80080) were purchased from Abcam (Cambridge, MA, USA). Recombinant human M-CSF, GM-CSF and murine M-CSF were purchased from PeproTech (Rocky Hill, NJ, USA). Human High Oxidized Low Density Lipoprotein (770252-7), Human DiI High Density Lipoprotein (770330-9) and Human High Density Lipoprotein (770300-4) were purchased from Kalen Biomedical (Germantown, MD, USA). AF-647-conjugated anti-His Ab (652513) and AF-647-conjugated mouse IgG1 Ab (400155) were purchased from Biolegend (San Diego, CA, USA). Human Fc block (5624219) and mouse Fc block (553142) were purchased from BD Biosciences (San Jose, CA, USA). PolymorphPrep was purchased from Axis-Shield (Norway). Paraformaldehyde (PFA; 16% wt/vol) was purchased from Electron Microscopy Sciences (Hatfield, PA, USA). Dako mounting medium was from Agilent Technologies (Santa Clara, CA, USA). Prolong Diamond Antifade Mountant was purchased from Life Technologies (Carlsbad, CA, USA). Accutase (07922) was purchased from Stemcell technologies (San Diego, CA, USA). RNeasy® Plus Mini Kit was purchased from Qiagen Canada (Toronto, ON, Canada). All cell culture media, and buffer solutions, unless mentioned otherwise, were purchased from Wisent (St-Bruno, QC, Canada).

### Expression and Purification of NSlit2, CSlit2, and ΔD2-Slit2

Bio-active truncated NSlit2 was purified from the conditioned medium of transfected HEK293-6E cells using Fractogel-cobalt column purification as previously described^21,23^ ΔD2-Slit2 (ΔD2), a Slit2 mutant which lacks the D2 domain required to bind Robo-1 was similarly purified^23^. CSlit2, an inactive fragment comprising amino acids 1268–1525 of Slit2, was cloned into the pTT28 vector using NheI BamHI restriction sites, and purified as described for NSlit2 and ΔD2^19,21,23,24^.

In all macrophage assays, NSlit2, CSlit2 and ΔD2 were diluted in serum-free cell culture medium (vehicle). Equimolar concentrations (30 nM) of NSlit2, CSlit2 and ΔD2 were used. In some experiments, Robo1N was used to block the binding of NSlit2 to Robo-1^21–23^. Robo1N and NSlit2 were incubated at a 3:1 molar ratio for 30 min, prior to adding to macrophages.

### Cell Isolation and culture

Peripheral blood mononuclear cells (PBMC) were isolated from the blood of healthy volunteers using PolyMorphPrep gradient separation solution according to the manufacturer’s instructions. Monocytes were incubated for 7–10 days with GM-CSF (50 ng/ml) or M-CSF (50 ng/ml) in RPMI 1640 supplemented with 10% heat-inactivated fetal bovine serum (FBS) and 1% penicillin/streptomycin/gentamicin.

Murine bone-marrow-derived macrophages (BMDM) were grown in whole-marrow cultures derived from wild-type (WT) and CD36^−/−^ mice on C57BL/6 background (Jackson Laboratories, USA) as well as from WT, Rac1^−/−^ and Rac2^−/−^ mice on a mixed B6/SV129 background^27,28^. BMDM were cultured in DMEM supplemented with recombinant murine M-CSF (50 ng/ml), 10% FBS and 1% penicillin/streptomycin/gentamicin. Murine RAW 264.7 macrophages were grown in DMEM supplemented with 10% FBS and 1% penicillin/streptomycin/gentamicin. DLD-1 cells were grown in RPMI 1640 supplemented with 10% FBS and 1% penicillin/streptomycin/gentamicin.

### RNA isolation, reverse transcription (RT) and polymerase chain reaction (PCR)

Total RNA was extracted using TRIzol Reagent according to the manufacturer’s instructions. cDNA was generated by reverse transcription (RT) using SuperScript III Reverse Transcriptase. Primer sequences used were: human Robo-1 (accession number NM_002941.3), sense 5′-CTATCGGCCATCTGGAGCCAAC-3′, and antisense 5′-GGAACAAGAAAGGGAATGACCACG-3′ and human GAPDH (accession number NM_002046), sense 5′-GAAGGTGAAGGTCGGAGTC-3′, and antisense 5′-AATGAAGGGGTCATTGATGGC-3′.

### Quantitative PCR (qPCR)

Total RNA was extracted using the RNeasy Plus Mini kit according to the manufacturer’s instructions. RNA concentration was measured using a NanoDrop 2000c Microvolume Spectrophometer. cDNA was generated by RT using SuperScript IV VILO Master Mix. qPCR was determined using POWER SYBR Green Master Mix on an Applied Biosystems StepOnePlus Real-Time PCR System. Primer sequences used were: mouse Robo-1 (accession number NM_019413), sense 5′-CCTTCAGACCTGATCGTCTCC-3′, antisense 5′-TGAGCGCGGGTCATCTTTG-3′ and mouse GAPDH (accession number NM_008084), sense 5′-AGGTCGGTGTGAACGGATTTG-3′, antisense 5′-TGTAGACCATGTAGTTGAGGTCA–3′^51,52^.

### Immunoblotting

Macrophage lysates were obtained using lysis buffer comprising 1× RIPA buffer supplemented with 1× protease inhibitor cocktail. Proteins were separated by SDS-PAGE and immunoblotting performed using anti-Robo-1 at 0.4 µg/ml or anti-β-actin antibodies as previously described^23^.

### Flow cytometry

Human M-CSF-induced macrophages and DLD-1 cells were washed then lifted with 10 mM EDTA (pH 8). Cells were incubated with LIVE/DEAD™ Fixable Violet viability dye for 15 min on ice followed by fixation with 4% paraformaldehyde (PFA). Cells were permeabilized with permeabilization buffer and incubated with human Fc block (10 µg/ml) for 15 min^53^. Next, cells were incubated with anti-Robo-1 Ab used at 10 µg/ml for 30 min. Cells were washed then incubated with AF-594-conjugated donkey anti-rabbit Ab used at 1 µg/ml for 15 min. All labelling steps were performed on ice. Samples were acquired using an LSRII analytical flow cytometer (BD Biosciences-US) with FACSDiva software (BD Biosciences-US) and analyzed with FlowJo v10 (BD Biosciences-US).

### Immunofluorescent labeling

Human macrophages, RAW 264.7 cells and WT BMDM were fixed with 4% PFA and incubated with mouse anti-Robo-1 Ab or mouse IgG1 antibodies, both used at 20 µg/ml for 3 h. Cells were washed, then incubated with Dylight549-conjugated anti-mouse IgG secondary Ab and AF-647-conjugated wheat germ agglutinin. Representative XZ optical slices were obtained using spinning disk confocal microscopy.

To quantify binding of NSlit2 to the surface of murine macrophages, cells were incubated on ice with NSlit2 for 20 min, washed, incubated with mouse Fc block (2.5 µg/ml) in 5% donkey serum for 15 min, then incubated with AF-647-conjugated anti-His or AF-647-conjugated mouse IgG1 antibodies, both used at 20 µg/ml for 20 min. Cells were washed, fixed with 4% PFA, and incubated with FITC-conjugated wheat germ agglutinin and DAPI to visualize the plasma membrane and nucleus, respectively. Representative XZ optical slices were obtained using spinning disk confocal microscopy. In some experiments, cells were blocked with anti-CD36 Ab or anti-IgG1 Ab both used at 2 µg/ml.

### OxLDL uptake assays

Murine RAW 264.7 cells, primary human macrophages, and BMDM from WT, Rac1^−/−^ and Rac2^−/−^ mice were grown on glass coverslips in 12-well plates and incubated with vehicle, NSlit2, or CSlit2 at 37 °C for 10 min. In some experiments, NSlit2 was pre-incubated with blocking Robo1N for 30 min prior to adding to cells. In other experiments, macrophages were incubated with anti-CD36 Ab or anti-IgG1 Ab at 37 °C for 30 min, prior to incubation with vehicle, NSlit2, or CSlit2. Cells were then incubated with oxLDL (50 µg/ml) for 24 h^54,55^. Lipid droplets were labeled using the fluorescent, neutral lipid probe, BODIPY (10 µg/ml) for 15 min. AF-647-conjugated wheat germ agglutinin was used to label the plasma membrane. Slides were imaged and representative XZ images acquired with an Olympus IX81 spinning disk confocal microscope (60× oil-immersion objective, NA 1.35), equipped with a Hamamatsu C9100-13 EM-CCD camera (Photometrics, Tucson, AZ). In some experiments, cells were lifted with Accutase after labeling with BODIPY and samples were acquired using a Gallios flow cytometer (Beckman Coulter-US) with Kaluza Acquisition software (Beckman Coulter-US) and analyzed with FlowJo v10 (BD Biosciences-US).

To quantify lipid content, an Amplex Red Cholesterol Assay Kit was also utilized. Human macrophages were grown in 96-well plates and incubated with vehicle, NSlit2, or CSlit2 at 37 °C for 10 min, then additionally with oxLDL (50 µg/ml) for 24 h. Amplex Red Cholesterol Assay Kit was used according to the manufacturer’s protocol.

In separate experiments, human macrophages were grown on glass coverslips in 12-well plates and incubated with vehicle, NSlit2, CSlit2, or ΔD2 at 37 °C for 10 min. Cells were then incubated with DiI-oxLDL (20 µg/ml) at 37 °C for 20 min, washed, and any surface bound DiI-oxLDL that was not internalized was removed using a 1 min wash with trypsin (0.25%)^29^. Cells were washed, fixed, and labeled with AF-488-conjugated wheat germ agglutinin. Slides were imaged as described above. In some experiments, cells were lifted with Accutase after incubation with DiI-oxLDL and samples were acquired using a Gallios flow cytometer (Beckman Coulter-US) with Kaluza Acquisition software and analyzed with FlowJo v10 (BD Biosciences-US).

### Cholesterol efflux assay

Cholesterol efflux was measured in RAW264.7 cells with the cholesterol efflux assay kit according to the manufacturer’s instructions and using human HDL as the cholesterol acceptor. In brief, RAW264.7 macrophages were grown in 96-well plates, washed with cold serum-free DMEM and incubated with labeling reagent overnight. Next, cells were washed with serum-free DMEM and incubated with NSlit2, CSlit2, and ΔD2 for 20 h. Following these incubations, fluorescence (Ex/Em = 482/515) was measured in cell supernatants and cell lysates using a fluorescence intensity microplate reader. The percentage of Cholesterol Efflux (C) was calculated using the formula C = 100 × (Fluorescence Intensity of Media)/ (Fluorescence Intensity of Cell Lysate + Media).

### OxLDL binding assays

Primary human macrophages were grown on glass coverslips in 12-well plates and incubated with vehicle, NSlit2, or CSlit2 at 37 °C for 10 min, then with DiI-oxLDL (2 µg/ml) for 1 min. This early timepoint was chosen because at 1 min, DiI-labeled oxLDL is still bound to the cell surface, but has not yet been internalized^29^. Cells were then washed, fixed, and labeled with AF-647-conjugated wheat germ agglutinin. Representative XZ optical slices were obtained using spinning disk confocal microscopy.

### Assessment of oxLDL binding and F-actin distribution

WT BMDM were grown on glass coverslips in 12-well plates and incubated with vehicle, NSlit2, CSlit2 or ΔD2 at 37 °C for 10 min, then with AF-555-conjugated oxLDL (2 µg/ml) at 37 °C for 1 min followed by fixation. Actin was labeled with AF-488-conjugated phalloidin. Samples were mounted using Prolong Diamond Antifade Mountant. Representative XZ optical slices were obtained using spinning disk confocal microscopy. Representative XY optical slices were obtained using the Carl Zeiss ELYRA PS1 system equipped with an Axio Observer Z1 microscope, a 63× (1.4 NA) apochromat oil immersion objective, a 1.6× optovar, and an Andor iXon3 885 EM-CCD camera^29^. Three orientation angles of the excitation grid with five phases each were acquired for each z plane. Images were reconstructed using the structured illumination module in the ZEN Software package (Carl Zeiss).

In parallel experiments, BMDM were treated with vehicle, NSlit2, CSlit2 or ΔD2 as described above, then fixed and incubated with rat anti-mouse CD36 used at 2 µg/ml for 1 h followed by AF-647-conjugated anti-rat IgG. After permeabilization, cells were labeled with AF-488-conjugated phalloidin. Representative XZ optical slices were obtained using spinning disk confocal microscopy.

### Image acquisition and fluorescence intensity data analysis

Slides were visualized using Olympus IX81, Leica DMIRE2 or Leica DMi8 spinning disk confocal microscopes equipped with 40× (NA-1.3), 60× (NA-1.35) and 63× (NA-1.4) oil-immersion objectives, and a Hamamatsu C9100-13 EM-CCD camera (Photometrics, Tucson, AZ). Image acquisition was controlled by Volocity Software (Perkin-Elmer, Waltham, MA) and 10 random fields of view were acquired per sample. Quantitative image analysis was performed using Volocity Software or ImageJ Software (NIH, Bethesda, MA, USA). For analysis performed on Image J, Z-stacks were imported as OME TIFF files, converted to hyperstacks, and then to z-projections using “sum slices” settings. Regions of interest were drawn around each cell, and the average intensity calculated after subtracting the background^29,56^.

### Statistical analysis

All statistical tests and *p* values are reported in corresponding figure legends. Analysis of variance (ANOVA) followed by Tukey’s post hoc tests were performed using GraphPad Prism Statistical Software (San Diego, CA, USA) to analyze data from experiments with multiple comparisons, unless specified otherwise. In all other cases, the Student’s t-test was used. Data represent mean values ± SEM. *p* < 0.05 was considered statistically significant.

## Supplementary Information

Supplementary Figures.

## Data Availability

The datasets generated during and/or analyzed during the current study are available from the corresponding author on reasonable request.
