# Supplementary material for: The neurorepellent, Slit2, prevents macrophage lipid loading by inhibiting CD36-dependent binding and internalization of oxidized low-density lipoprotein
Source: Sci Rep. 2021 Feb 11;11:3614. doi: 10.1038/s41598-021-83046-x (PMC7878733; doi:10.1038/s41598-021-83046-x)
Supplement: Supplementary file 1 — Supplementary Figures. [file 41598_2021_83046_MOESM1_ESM.pdf]

# THE NEUROREPELLENT, SLIT2, PREVENTS MACROPHAGE LIPID LOADING BY INHIBITING CD36-DEPENDENT BINDING AND INTERNALIZATION OF OXIDIZED LOW-DENSITY LIPOPROTEIN.

**Bushra Yusuf**[1,2,+], **Ilya Mukovozov**[3,+], **Sajedabanu Patel**[1], **Yi-Wei Huang**[1], **Guang Ying Liu**[1], **Emily C. Reddy**[4], **Marko Skrtic**[1], **Michael Glogauer**[5] and **Lisa A. Robinson**[1,2,6,*].

[1]Program in Cell Biology, The Hospital for Sick Children Research Institute, Toronto, ON M5G 1X8, Canada
[2]Institute of Medical Science, University of Toronto, Toronto, ON M5S 2Z9, Canada
[3]Department of Dermatology and Skin Science, University of British Columbia, Vancouver, BC, Canada
[4]Program in Developmental and Stem Cell Biology, The Hospital for Sick Children Research Institute, Toronto, ON M5G 1X8, Canada
[5]Faculty of Dentistry, University of Toronto, Matrix Dynamics Group, Toronto, ON M5G 1G6, Canada
[6]Department of Paediatrics, University of Toronto, Toronto, ON M5G 1X8, Canada
*Correspondence: lisa.robinson@sickkids.ca
[+] These authors contributed equally to this work

Supplementary Figures 1-3

Supplementary Figure 1. Human and murine macrophages express the Slit2 receptor, Robo-1.

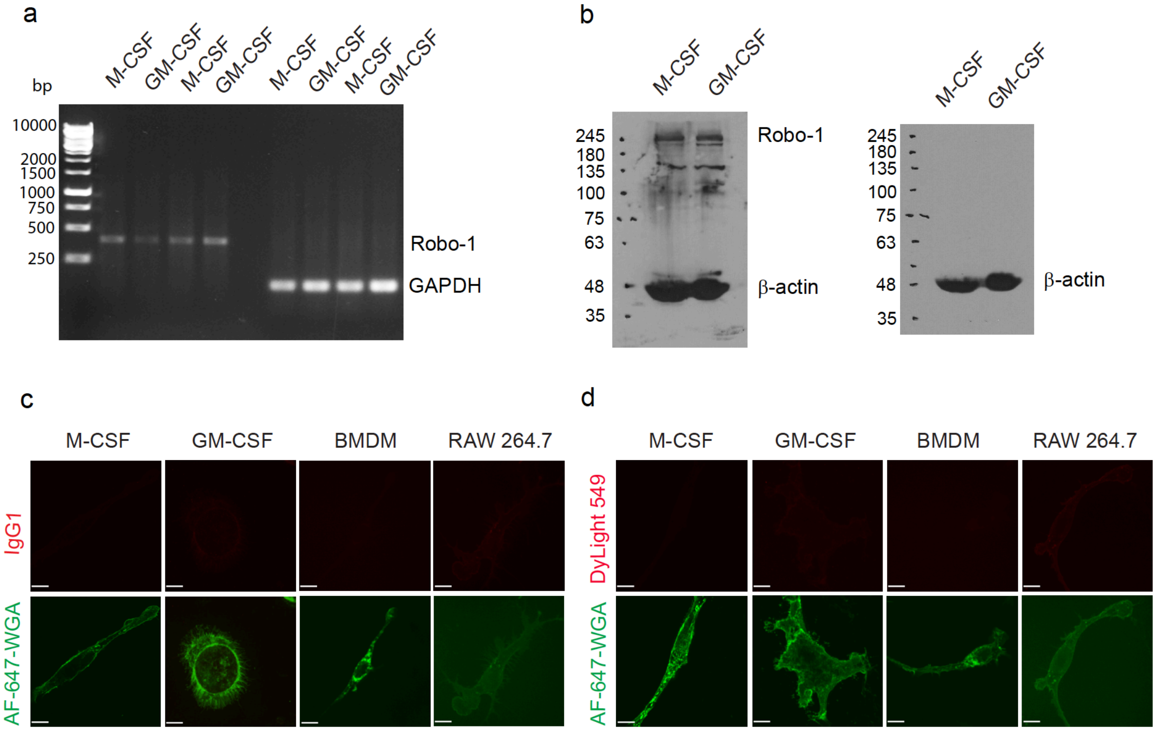


**Supplementary Figure 1**

**Human and murine macrophages express the Slit2 receptor, Robo-1.**

(**a**) PCR gel lanes 1-4 show Robo-1 in human M-CSF- and GMCSF- induced macrophages, as indicated. Lanes 5-8 show GAPDH in human M-CSF- and GM-CSF-induced macrophages, as indicated.

(**b**) (Left) Immunoblot lanes 1 and 2 show Robo-1 in human M-CSF- and GM-CSF-induced macrophages, respectively. (Right) The same immunoblot was stripped and re-probed for clearer visualization of the ß-actin bands.

(**c**) Macrophages were fixed and incubated with an IgG isotype control primary Ab, followed by Dylight549-conjugated secondary Ab (red) and AF-647-conjugated wheat germ agglutinin (pseudocoloured green). Cells were imaged using a spinning disk confocal microscope at 63x magnification. Scale bar, 10 µm.

(**d**) Macrophages were fixed and incubated with Dylight549-conjugated secondary Ab (red) and AF-647-conjugated wheat germ agglutinin (pseudocoloured green). Cells were imaged using a spinning disk confocal microscope at 63x magnification. Scale bar, 10 µm.

Supplementary Figure 2. NSlit2 inhibits uptake of oxLDL by macrophages.

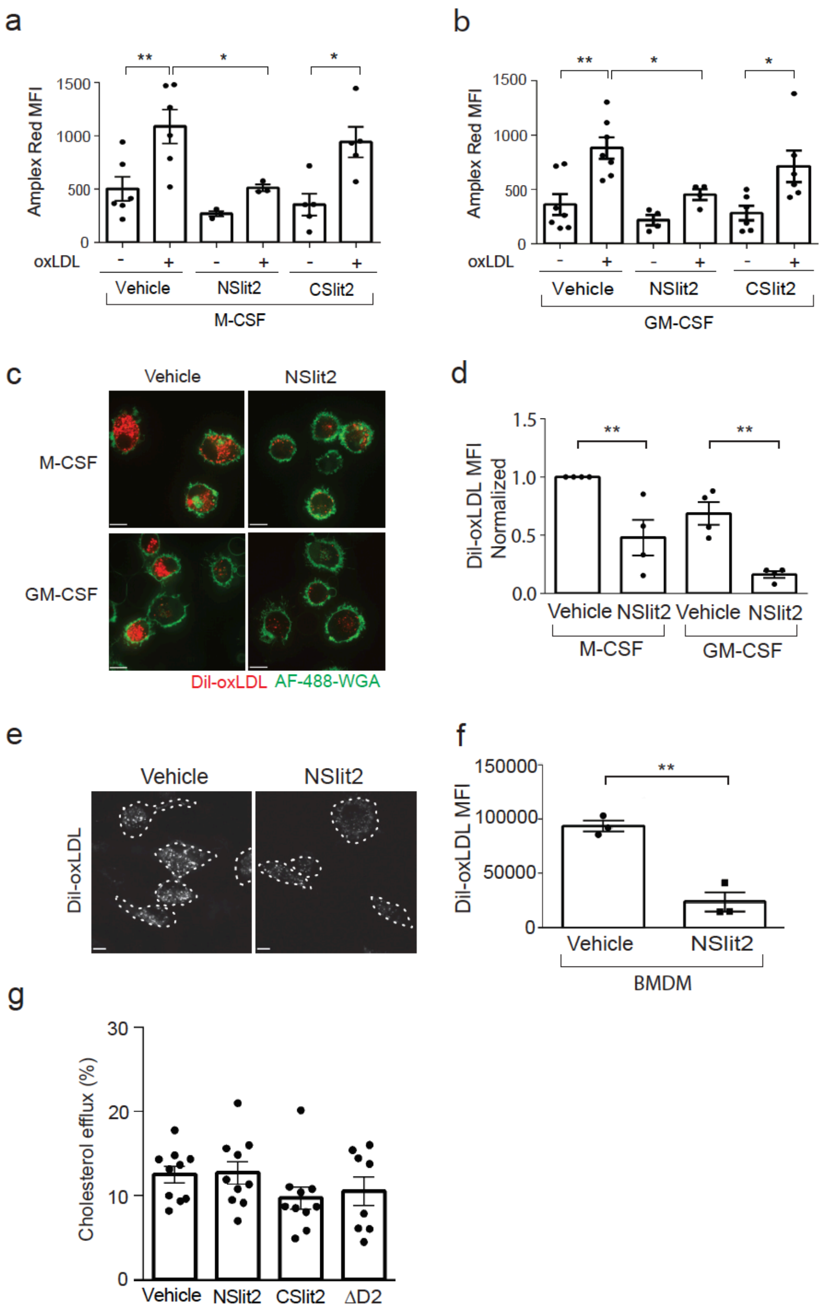


**Supplementary Figure 2**

**NSlit2 inhibits uptake of oxLDL by human and murine macrophages.**

(**a**), M-CSF-induced human macrophages were incubated with vehicle, NSlit2 or CSlit2, then incubated with oxLDL for 24 h. Internalized oxLDL was quantified using the Amplex Red Cholesterol Assay Kit. Data are the mean ± SEM of 3-6 independent experiments.

(**b**), Experiments were performed as in (a) using GM-CSF-induced human macrophages. Data are the mean ± SEM of 5-8 independent experiments.

(**c**), M-CSF- or GM-CSF-induced human macrophages were incubated with vehicle or NSlit2 then with DiI-labeled oxLDL (red) for 20 min. Cells were fixed and labeled with AF-488-conjugated wheat germ agglutinin (green). Representative images were obtained using a spinning disk confocal microscope at 60x magnification. Scale bar, 10 µm.

(**d**), Quantification of (c). DiI-oxLDL MFI was quantified using ImageJ Software. For each experimental condition, 20-40 cells were analyzed from 4 independent experiments. **$p<0.01$ was determined with one-way ANOVA using Tukey's post-hoc test.

(**e**), WT BMDM were incubated with vehicle or NSlit2, then incubated with DiI-oxLDL for 20 min. Representative images were obtained using a spinning disk confocal microscope. DiI- labeled oxLDL puncta are shown pseudocoloured white and cell borders are indicated via dashed lines. Scale bar, 10 μm.

(**f**), Quantification of (e). DiI-oxLDL MFI was measured using ImageJ Software. For each experimental condition, 40-50 cells were analyzed. Data are the mean ± SEM of 3 independent experiments. **$p<0.01$ was determined using an unpaired t-test.

(**g**), Cholesterol efflux from RAW264.7 macrophages was determined using a cholesterol efflux assay kit. Cells were first incubated with cholesterol labeling reagent then with vehicle, NSlit2, C-Slit or ΔD2. Following these incubations, cholesterol efflux onto the cholesterol acceptor, HDL, was measured using a fluorescent microplate reader. Data are the mean ± SEM of 8 to 10 independent experiments. Comparisons between groups were made with one-way ANOVA using Tukey's post-hoc test. All comparisons were ns.

For (**a**) and (**b**) *$p<0.05$, **$p<0.01$, were determined by one-way ANOVA using Holm-Sidak's post hoc test.

Supplementary Figure 3. NSlit2-mediated inhibition of oxLDL uptake by macrophages is CD36- and Rac1-dependent.

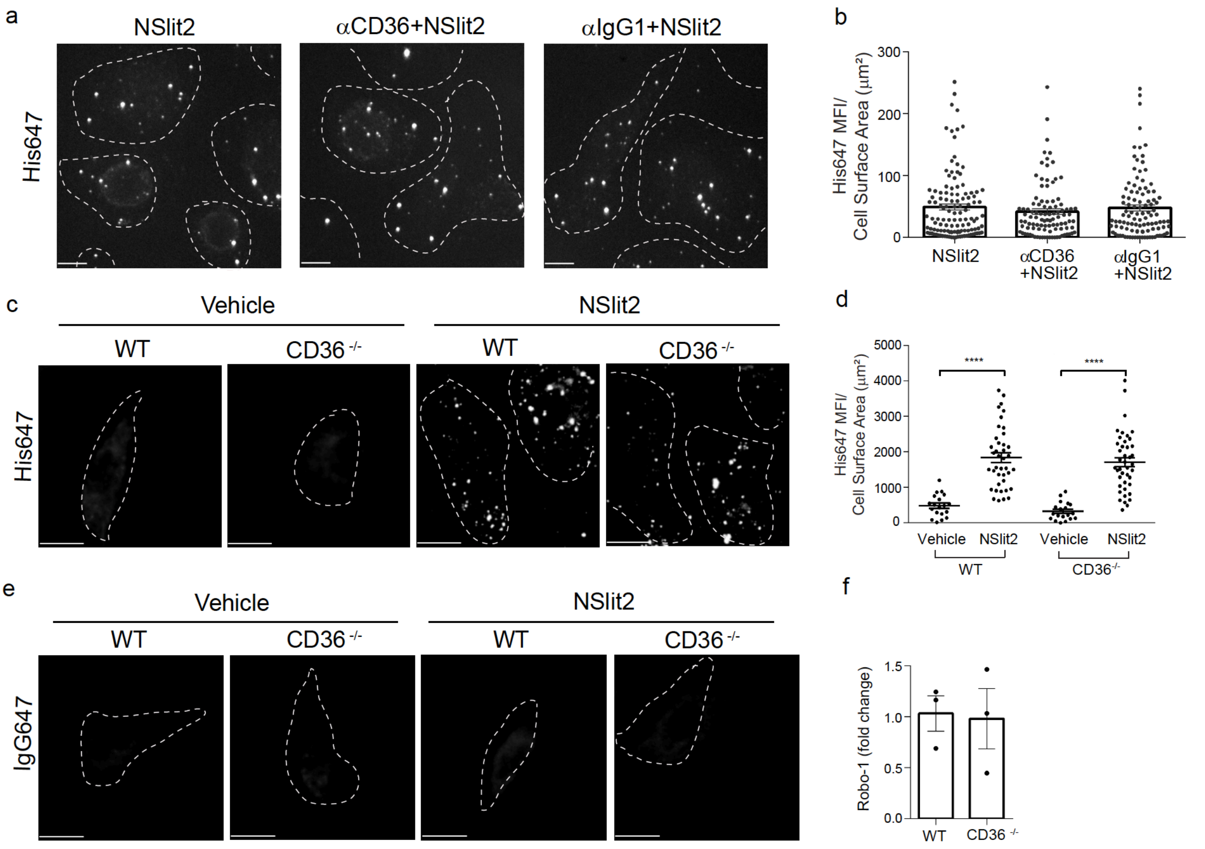


**Supplementary Figure 3**

**NSlit2-mediated inhibition of oxLDL uptake by macrophages is CD36- and Rac1-dependent.**

(**a**), Human M-CSF-induced macrophages were incubated in blocking buffer containing anti-CD36 Ab (α-CD36) or anti-IgG1 isotype control Ab (α-IgG1) at 4°C. Cells were then incubated with NSlit2 at 4°C followed by incubation with AF-647-conjugated anti-His Ab at 4°C. Cells were fixed and images were acquired using a spinning disk confocal microscope. Dashed lines indicate cell borders. Scale bar, 10 μm.

(**b**), Quantification of (a). His647 MFI was measured using Volocity Software and 30-40 cells were analyzed for each experimental condition. Individual dots correspond to single cells. Mean ± SEM from 3 independent experiments. Comparisons were made by one-way ANOVA using Tukey's post-hoc test and determined to be not significant.

(**c**), BMDM from wild-type (WT) and $CD36^{-/-}$ mice were incubated with vehicle (DPBS++) or NSlit2 at 4°C followed by incubation with AF-647-conjugated anti-His Ab at 4°C. Cells were fixed and representative images were acquired using a spinning disk confocal microscope. Dashed lines indicate cell borders. Scale bar, 10 μm.

(**d**), Quantification of (c). His647 MFI was measured using Volocity Software and 20-30 cells were analyzed for each experimental condition. Mean ± SEM from 4 independent experiments. Not significant and ****p<0.0001 were determined by one-way ANOVA using Tukey's post-hoc test.

(**e**), Experiments were performed as in (c) using AF-647-conjugated mouse IgG Ab. Dashed lines indicate cell borders. Scale bar, 10 µm.
(**f**), Total RNA was extracted from WT and CD36$^{-/-}$ BMDM and quantitative PCR was performed using specific primers for mouse Robo-1 and GAPDH. The fold change is displayed relative to the reference transcript, GAPDH. Data are the mean ± SEM from 3 independent experiments. A two-tailed, t-test was completed based on the delta $C_T$ measurements of the Robo-1 $C_T$ subtracted from the GAPDH $C_T$.
